# Experimental Evaluation of Quantum Dots and Antibodies Conjugation by Surface Plasmon Resonance Spectroscopy

**DOI:** 10.3390/ijms232012626

**Published:** 2022-10-20

**Authors:** Anton Popov, Viktorija Lisyte, Asta Kausaite-Minkstimiene, Eiva Bernotiene, Almira Ramanaviciene

**Affiliations:** 1 Department of Immunology, State Research Institute Centre for Innovative Medicine, Santariskiu Str. 5, LT-08406 Vilnius, Lithuania; 2 NanoTechnas—Center of Nanotechnology and Materials Science, Institute of Chemistry, Faculty of Chemistry and Geosciences, Vilnius University, Naugarduko Str. 24, LT-03225 Vilnius, Lithuania; 3 Department of Regenerative Medicine, State Research Institute Centre for Innovative Medicine, Santariskiu Str. 5, LT-08406 Vilnius, Lithuania; 4 Department of Chemistry and Bioengineering, The Faculty of Fundamental Sciences, Vilnius Gediminas Technical University, Vilnius-Tech, LT-10223 Vilnius, Lithuania

**Keywords:** surface plasmon resonance, quantum dots, antibody, conjugation, CD44

## Abstract

The application of antibody-functionalized quantum dots (QDs) in different areas has been widely described in the literature. However, a standard routine method for obtaining information on the conjugation efficiency of QDs with antibodies in terms of the interaction of the functionalized QDs with a specific antigen is still lacking. Herein, surface plasmon resonance (SPR) spectroscopy is proposed for this purpose. Gold-coated SPR sensor disks were modified with a self-assembled monolayer of 11-mercaptoundecanoic acid, and carbodiimide cross-linker chemistry was used to covalently immobilize the CD44 biomarker on the premodified surface (Au/CD44). Meanwhile, QDs functionalized with amine-derivatized polyethylene glycol (PEG) (QDs-NH_2_) were chosen for conjugation with antibodies because of their low non-specific adsorption on the Au/CD44 surface. Prior to conjugation, the surface binding capacity (*B_max_*) and equilibrium dissociation constant (*K_D_*) of the specific antibodies against CD44 (anti-CD44) were found to be 263.32 ± 2.44 m° and 1.00 × 10^−7^ ± 2.29 × 10^−9^ M, respectively. QDs-NH_2_ and anti-CD44 were conjugated at their initial molar ratios of 1:3, 1:5, 1:10 and 1:12. SPR measurements showed that the conjugates (QDs-anti-CD44) prepared using 1:10 and 1:12 molar ratios interacted comparably with immobilized CD44 biomarkers. The equilibrium angles in the case of 10- and 12-fold concentrations of anti-CD44 were calculated to be 60.43 ± 4.51 and 61.36 ± 4.40 m°, respectively. This could be explained by the QDs-NH_2_ and anti-CD44 having a similar surface loading (about four molecules per QDs-NH_2_) and similar hydrodynamic diameters, which were 46.63 ± 3.86 and 42.42 ± 0.80 nm for the 1:10 and 1:12 ratios, respectively. An initial QDs-NH_2_: anti-CD44 molar ratio of 1:10 was chosen as being optimal. SPR spectroscopy proved to be the right choice for QDs-anti-CD44 conjugation optimization, and can be used for the evaluation of conjugation efficiency for other nanostructures with various bio-recognition molecules.

## 1. Introduction

Quantum dots (QDs) are semiconductor nanomaterials that possess unique optical and electronic properties [1]. The bright emission and narrow wavelength bandwidth with broad absorption spectra [2] make QDs a perfect candidate as a label for multiplexed assay-based immunosensors [3]. Moreover, their high surface-to-volume ratio and tunable surface chemistry offer routes for manipulating the interactions of QDs with other molecules. It is possible to synthesize hydrophobic QDs as well as QDs with various hydrophilic coatings such as polymers, linker molecules, silanes, and various encapsulants such as liposomes and polymeric microbeads [4]. The large selection of QD surface coatings provides a choice of different methods of conjugation with antibodies, which can be grouped into covalent or non-covalent, and which provide random or site-directed antibody orientation [5]. Covalent conjugation provides strong irreversible binding, but the antibodies are randomly oriented. Additionally, conformational changes are possible [4].

Conjugates of QDs and antibodies have been widely applied in diagnostic [6], drug delivery [7], and tissue targeting and imaging [8], wherein the analytical performance of manufactured assays varied in accordance with binding efficiency to specific antigens, which depends on antibody orientation and loading density on the QDs [9]. Various methods, including ellipsometry [10], atomic force microscopy [11], mass spectrometry [12], and neutron reflectometry [13], can be used for the evaluation of antibody orientation, binding capacity, and coverage on the planar surface. However, application of these methods for such purposes is difficult or impossible when the evaluation of antibody deposition on nanostructures (NSs) is required. It has previously been shown that the efficiency of antibody conjugation to QDs can be evaluated prior to measurements using electrophoresis [14,15] and dynamic light scattering (DLS) [9,16,17]. In addition, DLS and nanoparticle tracking analysis (NTA) can be used to evaluate the interaction of NSs and antibody conjugates with free antigen in solution by tracking the change in size [18]. However, the polydispersity index of NSs must be low enough to use such a technique. Additionally, the ratio of QDs to antibodies in the conjugates can be calculated by measuring the amount of antibodies that have not been loaded onto the QD surface using ELISA with secondary antibodies [19]. In addition, specific methods can be used, the application of which depends on the type of NSs. For instance, the conjugation of QDs with antibodies was estimated by comparing fluorescence intensity after conjugation [20]. Radio-labeling of antibodies makes it possible to quantify the ratio of antibodies with NSs [21]. Time-of-flight secondary ion mass spectrometry can also be applied for the investigation of NS functionalization and interaction of their conjugates with proteins [22]. However, these methods mainly confirm the success of bioconjugation from changes in the physical parameters of the conjugates.

One of the most widely used methods for the investigation of molecular binding kinetics is the surface plasmon resonance (SPR) technique [23]. SPR spectroscopy has been applied for the detection of various biomolecules [24], viruses [25], and cells [26,27] due to its high sensitivity. The main advantage of SPR is the monitoring of binding kinetics without labeling requirements [28]. The possibility of multiple uses of the SPR sensor disk in automatic mode [29], provided by the well elaborated surface regeneration procedure [30], permits the use of fast and resource-saving biomolecule monitoring principles. Moreover, the SPR analytical signal depends on the interaction of the conjugates under study with the immobilized specific antigen, and this process is recorded in real time. Thus, the optimization of conjugate preparation can be assessed by the ability to bind to the antigen. In addition, the possibility of recording not only the association phase, but also the dissociation phase, makes it possible for us to obtain more information about the kinetics of the interaction. This makes the SPR method a perfect candidate for evaluating the conjugation efficiency of QDs and antibodies by conjugate-specific pair interaction.

CD44 is a non-kinase transmembrane glycoprotein, also referred to as extracellular matrix receptor III (ECMR-III), P-glycoprotein 1 (Pgp-1), and HUTCH-1, that is widely expressed on the surface of vertebrate cells, especially cancer stem cells [31]. CD44 has been extensively studied with respect to its modulating ability in tumor progression, metastasis, and disease prognosis [32]. CD44 and its isoforms are overexpressed in various cancer types and are crucially involved in cancer development by interacting with certain ligands such as hyaluronic acid and osteopontin [33]. CD44 overexpression interferes with the cytotoxic effects of chemotherapeutic drugs in various types of cancer. Therefore, high CD44 expression is correlated with poor prognosis in cancer treatment [34]. Moreover, CD44, as a regulator of glucose and lipid homeostasis in metabolic tissue, is involved in the pathogenesis of chronic metabolic diseases such as diabetes and obesity [35].

Here, we investigate the application of SPR spectroscopy for the evaluation of the efficiency of QD and antibody conjugation. This was the first time such a detailed study had been used for such an evaluation. Despite the abundance of articles on the use of such conjugates for various purposes, figuring out the optimal conjugation conditions remains a challenge. As a rule, not enough attention has been paid to optimization, and conjugation has not been evaluated in terms of the further interaction of the conjugates with the antigen. Choosing the right immobilization strategy, optimizing conjugation, and evaluating non-specific interactions, it is possible to increase the sensitivity and accuracy of the analytic or imaging system. In this paper, we present the results of SPR spectroscopy to evaluate the effectiveness of QD and antibody conjugations, depending on the specific conjugates binding to the antigen. This application of SPR spectroscopy could open the way to assessing conjugation efficiency for other nanostructures and biomolecules. QDs coated with amine-derivatized PEG (QDs-NH_2_) were covalently conjugated with specific antibodies against CD44 (anti-CD44) using various ratios. For this purpose, the cross-linking agent bis(sulfosuccinimidyl)suberate (BS3) was applied. The CD44 biomarkers were covalently immobilized on the surface of gold-coated SPR sensor disks through self-assembled monolayers (SAMs) of 11-mercaptoundecanoic acid (MUA). The optimal ratio of QDs-NH_2_ to anti-CD44 for conjugation was determined by studying the interaction of the prepared conjugates with immobilized CD44 biomarker using SPR spectroscopy.

## 2. Results and Discussion

MUA SAM formed on the surface of the SPR sensor disk was used for covalent immobilization of the CD44 protein. The coupling of proteins through alkanethiols on gold is well suited to performing SPR measurements [36]. Then, carboxyl groups of MUA were activated with an EDC/NHS mixture. This covalent attachment method has been widely used in the literature [37]. The SAMs formed on the gold surface not only provide a wide range of possible functional groups and the opportunity for covalent attachment to occur between various molecules, they also reduce nonspecific adsorption on the gold surface, which is extremely important when working with real samples [38,39]. Moreover, alkanethiols are thermally stable, can be used in wide pH range, and their SAMs form easily on gold via chemisorption [40].

**Figure 1:**
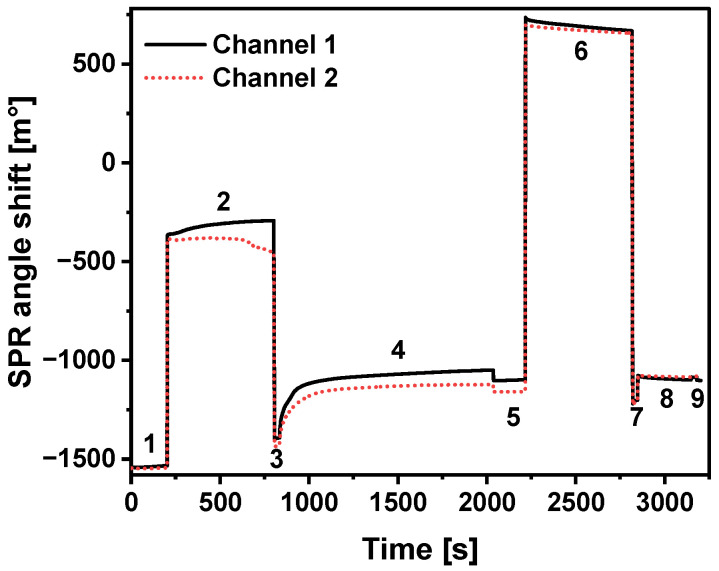
SPR study of CD44 immobilization in both channels of the MUA-modified SPR sensor disk. (1, 3, 5, 7—injection of acetate buffer, pH 4.5; 2—activation of MUA carboxyl groups with an EDC/NHS mixture; 4—immobilization of CD44; 6—deactivation of remaining activated carboxyl groups by 1 M ethanolamine solution; 8—injection of regeneration solution (50 mM NaOH and 0.5% SDS), 9—injection of PBS solution). The SPR angle shift for calculating the surface CD44 concentration was taken as the difference between the analytical signals at the beginning of CD44 immobilization (4) and at the end of the washing step (5).

The sensogram of CD44 covalent immobilization on MUA SAM in both channels is presented in Figure 1. It is obviously seen that SPR angle increases after injection of CD44 solution and during further incubation. The full kinetics of CD44 immobilization could be seen during the 4th stage of immobilization. Stable SPR angle, which signaling about reached saturation, was registered after approximately 15 min. The SPR angle shift for immobilization of CD44 was equal to 265 ± 9 m°, and corresponds to 2.17 ± 0.07 ng/mm^2^ surface concentration. The linear relationship (Section 3.7) between the SPR angle shift and the amount of immobilized CD44 protein was used to calculate the mass concentration on the surface.

The two types of QDs with carboxyl groups (QDs-COOH) and amino groups (QDs-NH_2_) were selected to perform further covalent conjugation with specific antibodies. The surface of QDs-NH_2_ was functionalized by amine-derivatized PEG. This modification not only provides the opportunity to perform the covalent immobilization of biomolecules, but can also potentially reduce non-specific interaction with premodified surfaces [4].

**Figure 2:**
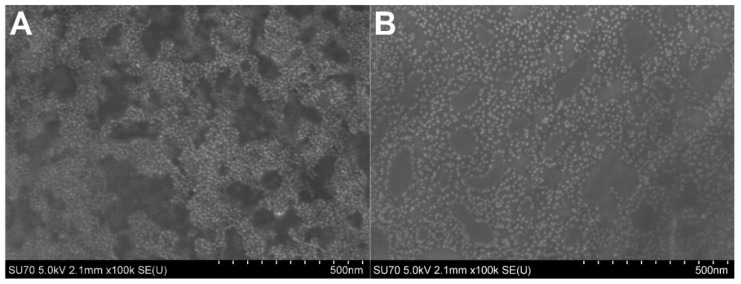
SEM images of (**A**) QDs-COOH and (**B**) QDs-NH_2_.

The chosen QDs were characterized using the scanning electron microscopy (SEM) technique (Figure 2). The shape of QDs-NH_2_ (Figure 2B) was spherical, with a diameter of 8.96 ± 0.85 nm, where QDs-COOH were more spheroidal (Figure 2A). The organic coating of QDs complicated the increase in magnification of images by SEM and the application of a higher voltage. Moreover, the hydrodynamic diameter (*D_H_*) of the QDs was measured using dynamic light scattering (DLS) (Table 1), and was about 30 nm for both of the QDs used. Both QDs were stable and not prone to aggregation.

**Figure 3:**
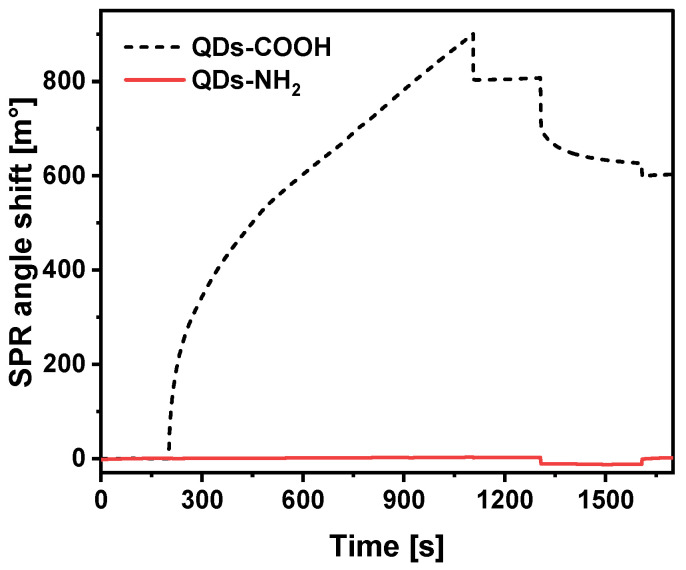
Non-specific adsorption of QDs-NH_2_ and QDs-COOH on the surface of Au/CD44.

The use of specific antibodies and QD conjugates, which possess high non-specific interaction with an analyte, could be complicated or even impossible due to adsorption of analyte molecules on free QD surface. Moreover, in case of a non-specific interaction between QDs and a particular protein, the possible adsorption of other similar molecules can be predicted, which can complicate the use of conjugates for analysis in real samples [4]. The interactions of QDs-COOH and QDs-NH_2_ with CD44 protein immobilized on the surface of Au/MUA were investigated (Figure 3). The 10 nM concentration of QDs was used for these experiments. Following baseline stabilization, 10 nM of QD solution was injected into the SPR cuvette. A small increase of about 2 m° was observed in the case of QDs-NH_2_. After the dissociation and subsequent regeneration procedure, the SPR signal almost returned to the level observed before injection of the QDs-NH_2_. Injection of 100 nM QDs-NH_2_ solution gave a similar result. In contrast, after QDs-COOH injection, a systematic intensive shift in the SPR signal was registered during further incubation. Moreover, a shift in the SPR signal of about 800 m° was registered after dissociation. Even further regeneration did not allow detachment of QDs-COOH from the surface of Au/CD44. This clearly illustrates high non-specific interaction of QDs-COOH with immobilized CD44 protein, while the adsorption of QDs-NH_2_ was negligible. According to these results, QDs-NH_2_ were selected for further conjugation with specific antibodies.

**Figure 4:**
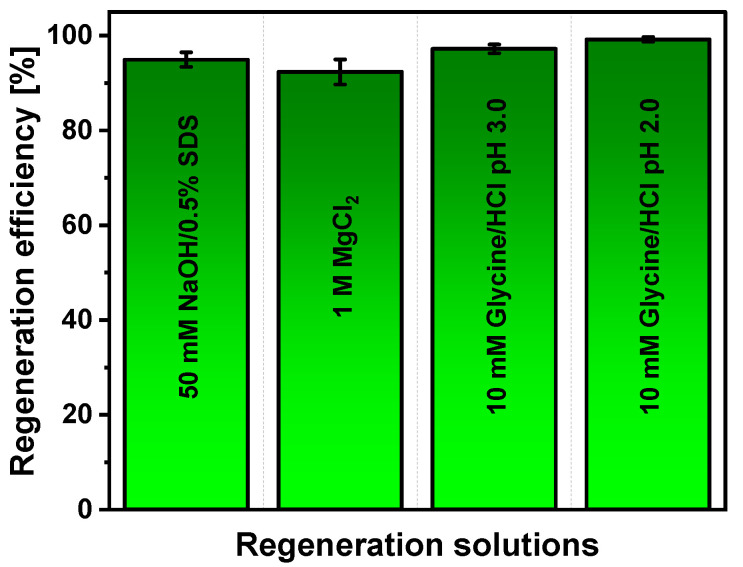
Dependence of Au/CD44/anti-CD44 complex dissociation and surface regeneration efficiency on the nature of the regeneration solution.

The regeneration procedure is important for multiple uses of SPR sensors. In the process of regeneration, the non-covalent bond and the interactions between the antibody paratope and the antigen epitope must be broken without affecting the characteristics of immune complex formation. The efficiency of the regeneration solution depends on the nature of bonding, since there are different options, such as interactions between Lewis acids and Lewis bases, the electrostatic force, van der Waals forces, and hydrogen bonds [41]. The results of regeneration solution selection are presented in Figure 4. Experiments were performed after incubation of Au/CD44 in 30 nM solution of anti-CD44 and further regeneration with a chosen solution.

It was found that all tested regeneration solutions provided regeneration efficiency higher than 92%, wherein the best performance (99.22 ± 0.49%) was registered using 10 mM glycine/HCl, pH 2. In the case of 50 mM NaOH/0.5% SDS, 1 M MgCl_2_, and 10 mM glycine/HCl, pH 3, the regeneration efficiency was equal to 94.95 ± 1.54, 92.34 ± 2.63, and 97.22 ± 0.91%, respectively. This illustrates that the sensing surface could be used for analysis, when the 10 mM glycine/HCl, pH 2, solution is applied for regeneration.

**Figure 5:**
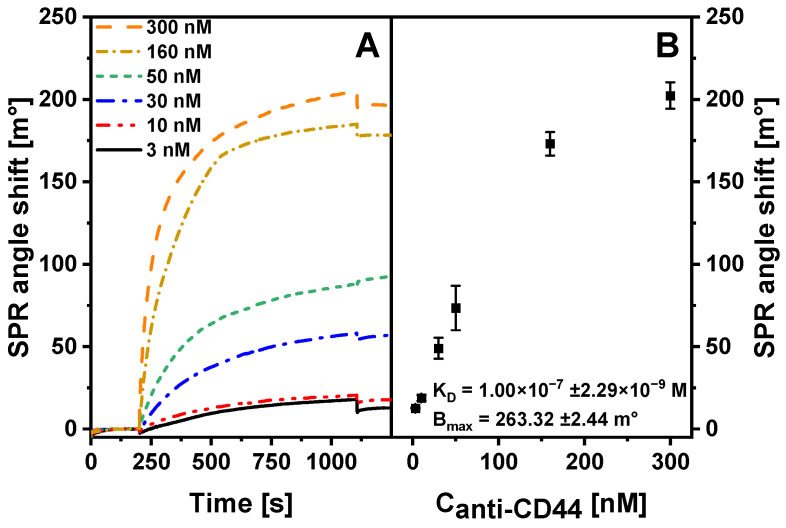
(**A**) SPR study of immune complex formation between immobilized CD44 protein and anti-CD44 antibody at concentrations of 3, 10, 30, 50, 160 and 300 nM. (**B**) The dependence of SPR response on concentration of anti-CD44 antibodies. The SPR response was calculated as the difference between the SPR signal at the end of the dissociation phase and at the beginning of the association phase.

An SPR kinetic study can provide useful insights into the molecular interactions [42] and antibody–antigen affinity binding [43], thus evaluating the suitability of an antibody for further conjugation, since a weak antibody–antigen interaction will also reduce the binding of nanoparticle–antibody conjugates. The interaction of anti-CD44 antibodies with immobilized CD44 protein was investigated (Figure 5). Anti-CD44 concentration varied from 3 to 500 nM. The SPR response was found to increase with increasing concentration of the injected anti-CD44 solution. SPR response using anti-CD44 concentration of 3 nM was 12.57 ± 0.39 m°, whereas the injection of 300 nM anti-CD44 solution yielded an SPR response of 202.34 ± 8.00 m°. Equilibrium kinetic parameters were then calculated to evaluate the affinity of anti-CD44. *K_D_* represents the strength of antibody–antigen association. A lower value of *K_D_* indicates a higher-affinity interaction. The *K_D_* value for the antibody interaction with antigen in solution is usually in the range from 10^−9^ to 10^−6^ M [44]. *K_D_* and *B_max_* were calculated to be 1.00 × 10^−7^ ± 2.29 × 10^−9^ M and 263.32 ± 2.44 m°, respectively. According to these data, the anti-CD44 antibody interacts strongly with immobilized CD44 protein, and should be suitable for conjugation with QDs-NH_2_.

Covalent attachment was chosen for the conjugation of anti-CD44 and QDs-NH_2_ using BS3 reagent. Amine-derivatized PEG on the surface of QDs-NH_2_ makes it possible to avoid the formation of non-specific interaction with antibodies providing covalent attachment. Water-soluble BS3 cross-linking agent is a widely used reagent whose N-hydroxysulfosuccinimide (NHS) ester groups are able to interact with the primary amines of the N-terminus region of polypeptides and the side chain of lysine residues to form amide bonds under pH 7–9 [45]. BS3 has previously been used for QD conjugation with antibodies, which were then successfully applied in living cells [46,47,48]. Moreover, the attachment of biomolecules through a cross-linker provides additional spatial freedom to the immobilized molecules and ensures the effective affinity interactions [4]. QDs-NH_2_: anti-CD44 molar ratios of 1:3, 1:5, 1:10, and 1:12 were tested to determine optimal conjugation conditions. No significant aggregation or leakage of QDs-NH_2_ was observed during conjugation. The obtained conjugates were collected, and their concentrations were determined according to QDs-NH_2_ calibration curve (Figure S1).

Conjugates were characterized by DLS (Table 1). It was found that the hydrodynamic diameter of conjugates was bigger than that of non-modified QDs-NH_2_. In the case of the 1:3 and 1:5 molar ratios of QDs-NH_2_ and anti-CD44, the *D_H_* increased from 30.21 ± 1.80 nm for QDs-NH_2_ to 35.31 ± 1.14 and 38.09 ± 1.62 nm for QDs-anti-CD44, respectively. The hydrodynamic diameters of conjugates prepared using initial QDs-NH_2_: anti-CD44 molar ratios of 1:10 and 1:12 were 46.63 ± 3.86 and 42.42 ± 0.80 nm, respectively, whereby *D_H_* changed by approximately 14 nm. A similar increase in *D_H_* (13.2 ± 1.0 nm) was observed in the case of antibody adsorption onto the surface of gold nanoparticles at pH 7.5 [9]. Moreover, the anti-CD44 antibody used in this work was immunoglobulin G, which has dimensions of 14 × 8.5 × 4.5 nm [49]. The theoretical increase in *D_H_* is dependent on the orientation of the antibodies, and can be from 9 to 28 nm, which corresponds to the “flat-on” and “head-on” orientations, respectively [9]. This observation suggests that, in our case, there is a mixed orientation of the antibodies, which would be expected when using covalent conjugation.

**Figure 6:**
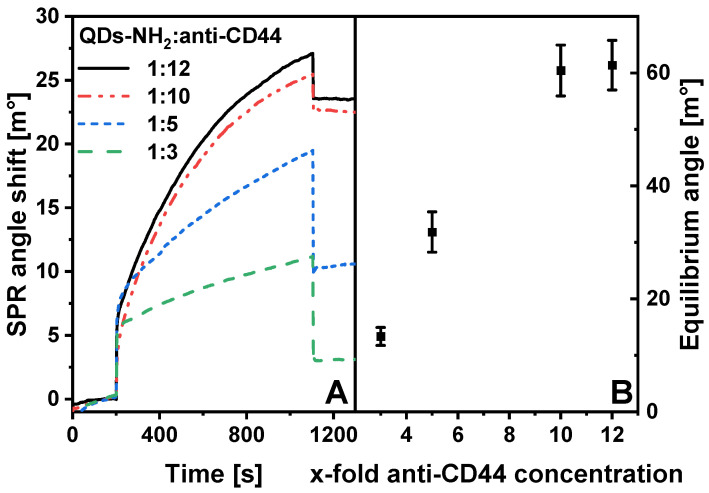
(**A**) SPR sensograms of the interaction of QDs-anti-CD44 conjugates prepared using QDs-NH_2_: anti-CD44 molar ratios of 1:3, 1:5, 1:10, and 1:12 with Au/CD44. (**B**) Dependence of the equilibrium angle on the x-fold concentration of anti-CD44.

Furthermore, the efficiency of QDs-NH_2_ and anti-CD44 conjugation was investigated by registering the interaction of immobilized CD44 protein with the injection of 5 nM solution of bioconjugates (Figure 6A). The interactions of QDs-anti-CD44 conjugates prepared using different initial QDs-NH_2_: anti-CD44 molar ratios were compared.

The results showed that the prepared conjugates successfully interacted covalently with immobilized CD44 on an MUA SAM premodified SPR sensor disk. The continuous upward shift of the SPR signal was registered during the association phase in all experiments. The lowest SPR angle shift was monitored in the case of the 1:3 molar ratio of QDs-NH_2_: anti-CD44. A 5-fold increase in the initial concentration of anti-CD44 compared to that of QDs-NH_2_ resulted in a significant increase in the SPR signal during the association phase. However, the SPR signal decreased significantly during dissociation, which may indicate the adsorption of conjugates onto the surface or non-specific interaction with the CD44 premodified SPR sensor disk. The SPR angle shifts observed during the interaction experiments of the conjugates, which were prepared using initial molar ratios of 1:10 and 1:12, were very similar to each other. The decrease in the SPR signal upon dissociation was moderate, indicating a specific interaction of bioconjugates with CD44 protein. Equilibrium angles of interactions were calculated, and their average values were plotted against an x-fold initial concentration of anti-CD44 (Figure 6B). In the case of a 5-fold concentration of anti-CD44, the equilibrium angle was 31.83 ± 3.58 m°, and was almost 2.4 times higher when compared with the equilibrium angle (13.34 ± 1.60 m°) calculated in the case of a 3-fold concentration of anti-CD44. The equilibrium angle was also almost half that of the analytical signals (60.43 ± 4.51 and 61.36 ± 4.40 m°) calculated for samples in which the initial QDs-NH_2_: anti-CD44 molar ratios were 1:10 and 1:12. It can be summarized that the interaction of QDs-anti-CD44 and immobilized CD44 protein was at the same level for samples that were obtained using 10- and 12-fold anti-CD44 concentrations.

Additional comparison of QDs-anti-CD44 conjugate formation efficiency on the basis of the evaluation of the concentration of antibodies unbound to QDs-NH_2_ was performed. The bioconjugates prepared using initial molar QDs-NH_2_: anti-CD44 ratios of 1:10 and 1:12 were evaluated. First, the direct ELISA format was used to prepare calibration curve (Figure S2) for the detection of anti-CD44 antibody concentration. The filtrates collected at the washing step of the QDs-anti-CD44 conjugates were then diluted, and the amount of unbound antibodies was calculated using a calibration curve. The ratio of QDs-NH_2_ to anti-CD44 in the conjugates was evaluated using the obtained results. Experimentally, it was found that about four anti-CD44 molecules bound to one quantum dot, regardless of with initial QDs-NH_2_: anti-CD44 molar ratios were used. These results are in agreement with the SPR results of the interaction of QDs-anti-CD44 with immobilized CD44 protein and DLS measurements. Similar hydrodynamic diameters were recorded for QDs-anti-CD44 formed at ratios of 1:10 and 1:12, which may indicate the same QDs-NH_2_ coverage by the antibodies. On the basis of these data, it was concluded that a QDs-NH_2_: anti-CD44 molar ratio of 1:10 is optimal for conjugation.

The study of conjugate-specific affinity interaction with immobilized antigen can provide additional information on the efficiency of conjugation. The CD44 biomarker was immobilized on the surface of the SPR sensor disk using initial concentrations of 75, 150, 300, and 600 nM. Next, the interaction of conjugates prepared using the optimal initial QDs-NH_2_: anti-CD44 ratio was studied. The results are shown in Figure S3. Decreasing the initial concentration of CD44 in the solution from 600 to 300 nM (and on the surface of SPR sensor disk after covalent immobilization) had no significant effect on the interaction with the added conjugates, which is most likely due to the fact that the surface density of CD44 decreased from only 2.17 to 1.94 ng/mm^2^, reducing the biomarker surface concentration. Further reduction of the initial CD44 concentration affected the amount of immobilized biomarker and its interaction with conjugates. The SPR response is reduced by 26 and 38% when using an initial CD44 concentration of 150 and 75 nM, respectively. The surface density in these cases decreased by more than 50%. Thus, a decrease in the amount of CD44 on the surface of the SPR disk leads to a decrease in the conjugate molecules interacting with immobilized biomarkers, but the signal is still recorded. This observation suggests that the prepared conjugates could interact with CD44 biomarkers distributed both closer and further away on the surface of the SPR sensor disk or on the surface of the affected cell.

**Figure 7:**
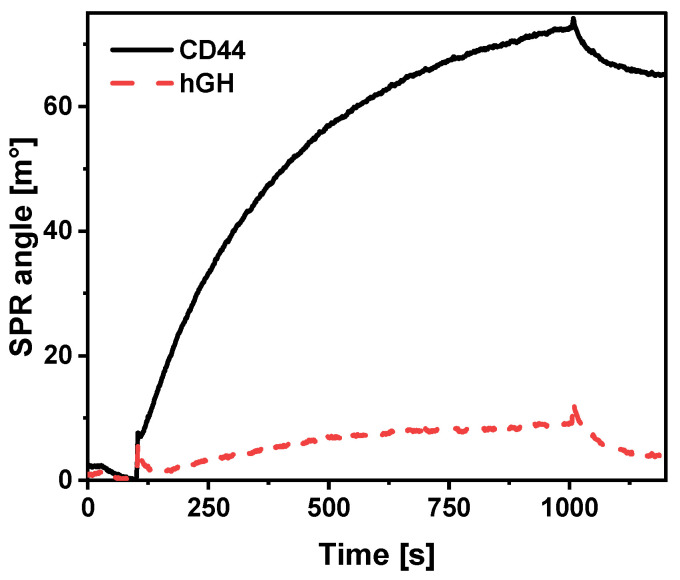
Interaction of QDs-anti-CD44 (QDs-NH_2_: anti-CD44 molar ratio 1:10) with CD44 and hGH immobilized on the surface of Au/MUA in different channels.

CD44 is expressed on a variety of cells, the surfaces of which are saturated with other molecules and biomarkers. The specific interaction must be provided to ensure that there are no false positive results. The specificity of the binding of QDs-anti-CD44 to immobilized CD44 was investigated. For this purpose, CD44 protein and human growth hormone (hGH) were immobilized in the different channels of the SPR cuvette. Immobilization was performed under the same conditions, as was described for the CD44 protein. hGH was chosen for comparison as a polypeptide with a molecular mass of 22 kDa [50], which is similar to the molecular mass of the CD44 protein used. A solution of 15 nM QDs-anti-CD44 conjugates (QDs-NH_2_: anti-CD44 molar ratio 1:10) was injected into the SPR cuvette and incubation was monitored for 900 s (Figure 7). The specificity was evaluated by comparing the changes in SPR signal of QDs-anti-CD44 interaction with Au/CD44 and Au/hGH. In the case of the immobilized CD44 protein, a continuous upward shift was observed. When QDs-anti-CD44 was injected into the channel with Au/hGH, an increase in the SPR signal was observed, but it was much less intense. Moreover, at the end of the dissociation phase, the SPR signal had almost returned to the Au/hGH level. The SPR response in the case of Au/CD44 was 65 m° and more than 16 times higher than that in the case of Au/hGH. This can partly be explained by the surface coating of the QDs with amine-derivatized PEG, which decreases non-specific interaction with immobilized hGH. Finally, it should be noted that prepared QDs-anti-CD44 conjugates specifically interact with the immobilized CD44 protein.

## 3. Materials and Methods

### 3.1. Materials and Reagents

Recombinant human CD44 biomarker (career free, His tag C-Termus), monoclonal mouse IgG clone # 2C5 (anti-CD44) and recombinant human growth hormone from *E. coli* (hGH) were obtained from R&D Systems (Abingdon, UK). Goat anti-mouse IgG Fc secondary antibody labelled with horseradish peroxidase (anti-HRP), qdot™ 605 ITK™ amino (PEG) quantum dots (QDs-NH_2_), qdot™ 655 ITK™ carboxyl quantum dots (QDs-COOH), bis(sulfosuccinimidyl)suberate (BS3) and 3,3′,5,5′-tetramethylbenzidine solution (TMB) were purchased from Thermo Fisher Scientific (Eugene, OR, USA). Hydrogen peroxide (H_2_O_2_), sulfuric acid (H_2_SO_4_), sodium hydroxide (NaOH), sodium dodecyl sulfate (SDS), 1-ethyl-3-(3-dimethylaminopropyl)carbodiimide hydrochloride (EDC), N-hydroxysuccinimide (NHS), hydrochloric acid (HCl), methanol (CH_3_OH), magnesium chloride (MgCl_2_) and bovine serum albumin (BSA) were obtained from Carl Roth GmbH & Co. (Karlsruhe, Germany). Absolute ethanol was received from Honeywell (Charlotte, NC, USA). Glycine, Tween-20 and 11-mercaptoundecanoic acid (MUA) were acquired from Sigma-Aldrich (Steinheim, Germany). Immersion oil (refractive index *n* = 1.518) was purchased from Cargille Laboratories (Cedar Grove, NJ, USA). 10 mM Phosphate-buffered saline, pH 7.4 (PBS), solution was prepared from tablets (Carl Roth GmbH & Co. (Karlsruhe, Germany)). 10 mM acetate buffer, pH 4.5, was made from sodium acetate trihydrate (CH_3_COONa·3H_2_O) and acetic acid (CH_3_COOH) obtained from Carl Roth GmbH & Co. (Karlsruhe, Germany). Ultra high-quality water was used for sample preparation.

### 3.2. Preparation and Characterization of QDs-Anti-CD44 Conjugates

Before modification, QDs-NH_2_ were centrifuged at 2000× *g* for 3 min to exclude aggregates. Then QDs-NH_2_ in PBS were mixed with BS3 reagent in a molar ratio of 1:1000 in LoBind microcentrifuge tubes (Eppendorf, Hamburg, Germany). The solution was stirred using tube revolver/rotator (Thermo Fisher Scientific, Waltham, MA, USA) for 30 min at room temperature. Excess of cross-linker was removed using a 100 K Nanosep centrifugal filter (Pall, Carolina, Puerto Rico). Washing procedure was performed three times using 500 µL of PBS. Concentrated solution was divided into several parts, each of which was mixed with anti-CD44 antibodies in molar ratios of 1:3, 1:5, 1:10, and 1:12. The prepared solutions were slowly stirred for 2 h at room temperature. Next, the addition of 100 μL 1 M glycine and further mixing for 15 min was performed. QDs-anti-CD44 conjugates were purified using a 300 K Nanosep centrifugal filter (Pall, Carolina, Puerto Rico) by ultrafiltration for at least 3 times with PBS. The filtrate was collected for further evaluation of antibody conjugation efficiency. Solutions of purified conjugates were kept before measurements at 4 °C.

The absorption of QDs-NH_2_ at 405 nm wavelength was registered using plate reader Infinite 200 PRO (Tecan, Zurich, Switzerland) and calibration curve of QDs-NH_2_ was established in the range from 5 to 80 nM. The shape of QDs was characterized using a high-resolution field emission scanning electron microscope SU-70 (Hitachi, Ibaraki, Japan) (FE-SEM). The hydrodynamic diameters of anti-CD44, QDs, and functionalized-QDs were evaluated using Zetasizer Nano ZS 3600 instrument (Malvern, UK). The hydrodynamic diameter was recorded in PBS.

### 3.3. The SAM Formation on Gold-Coated SPR Sensor Disk

The surface of gold-coated SPR sensor disks (SPR.BK7.AU, Kinetic Evaluation Instruments, Leusden, The Netherlands) was carefully cleaned before formation of MUA SAM. The sensor disks were kept in piranha solution (3:1 H_2_SO_4_ and 30% H_2_O_2_) for 5 min at 60 °C and further washed using ethanol and deionized water. Cleaned sensor disks were placed in a vessel with 1 mM MUA methanol-based solution. The formation of SAM was performed for 24 h. Modified sensor disks were additionally washed with ethanol and deionized water, and dried under N_2_ gas.

A drop of immersion oil was poured onto the surface of hemi-cylinder assembled on a slider, MUA-modified sensor disk (Au/MUA) was mounted on the top, and the slider was placed into the SPR instrument (Autolab Espirit, Metrohm Autolab BV, Leusden, The Netherlands). This instrument makes it possible to perform simultaneous SPR measurements in 2 separate channels (surface area in one channel—7.9 mm^2^). The steps of stabilization/rehydration of the MUA-modified surface of sensor disks were performed by incubating for around 45 min at 2 min intervals in 10 mM acetate buffer, pH 4.5, and in regeneration solution (50 mM NaOH and 0.5% SDS aqueous solution) until the baseline stabilized.

### 3.4. The Immobilization of CD44 Biomarker on the MUA Modified SPR Sensor Disk

Immobilization was performed in both channels of the SPR cuvette. First, 10 mM acetate buffer, pH 4.5, was used as coupling buffer, and was injected into the channels and mixed for 200 s. Activation of the carboxyl group was performed by keeping MUA SAM premodified SPR sensor disks in a solution (mixed at ratio 1:1) of freshly prepared 0.4 M 1-ethyl-3-(3-dimethylaminopropyl)carbodiimide hydrochloride (EDC) and 0.1 M N-hydroxysuccinimide (NHS) for 600 s. After rinsing the SPR cuvette, 600 nM of CD44 solution was injected, and covalent immobilization was carried out for 1200 s. Unbound CD44 molecules were removed from both channels during rinsing with the coupling buffer. Deactivation of the remaining activated carboxyl groups was performed by means of incubation in 1 M ethanolamine solution for 600 s. Steps of stabilization/rehydration were repeated to obtain a stable baseline. As a result, CD44 biomarker immobilized on surface of SPR sensor disk (Au/CD44) through amide bounds was prepared prior to performing further measurements.

### 3.5. Interaction of QDs-Anti-CD44 with Au/CD44

The SPR technique was applied for the characterization of specific anti-CD44 antibodies, selection of regeneration solution, evaluation of non-specific binding of QDs, and optimization of the molar ratio for the conjugation of QDs-NH_2_ and antibodies.

A stable baseline was reached upon incubation of Au/CD44 in PBS for 200 s. Immediately after QDs-anti-CD44 solution was injected and mixed for 900 s, injection was performed in one channel, and the other one was used as a reference, into which PBS solution was poured. Dissociation of the formed CD44: anti-CD44-QDs complex was performed by incubation in PBS solution for 200 s. The regeneration of the sensor disk surface (up to Au/CD44) was performed using 10 mM glycine/HCl, pH 2, solution, and further baseline was registered in PBS. The analytical signal was recorded as the difference between the measurement and the reference channels. The control experiments with anti-CD44 at various concentrations or QDs were performed under the same conditions. All dilutions were performed using PBS.

### 3.6. Spectrophotometric Determination of Anti-CD44 Antibody

Anti-CD44 concentration was measured in MaxiSorp plates from Thermo Fisher Scientific (Eugene, OR, USA) using the direct ELISA format. Briefly, 50 µL of anti-CD44 antibody at concentrations varying between 0.01 and 0.7 µg/mL was added to the wells. Incubation was performed for 2 h at 37 °C. The wells were washed 3 times with PBS containing 0.05% of Tween 20. Next, the blocking of the free remaining surface was performed by adding 200 µL of 2% BSA to each well and incubating for 1 h. The washing procedure was repeated, and then 200 µL of 100 ng/mL anti-HRP was added. After 2 h, the wells were washed and 100 µL of TMB solution was added. The reaction was stopped after 15 min with 100 µL of 1 M HCl. The absorption at 450 nm was measured, and a calibration plot was plotted. This calibration curve was used for the evaluation of the efficiency of covalent antibody immobilization on QDs-NH_2_.

### 3.7. Calculations

The change in refractive index caused by the binding of molecules to the SPR sensor disk surface results in a shift in the SPR angle. An SPR angle shift of 120 m° represents a change in surface mass concentration of about 1 ng/mm^2^. The surface mass concentration of CD44 was calculated on the basis of this linear relationship.

The percentage of regeneration efficiency of Au/CD44/anti-CD44-QDs was calculated as the ratio of the baselines of the SPR signal before the interaction between Au/CD44 and anti-CD44 antibodies, and after treatment with the regeneration solution.

The surface-binding capacity (*B_max_*) and equilibrium dissociation constant (*K_D_*) were determined by approximating the results of the association region of anti-CD44 interaction with Au/CD44 to a single-site interaction model [28]:

*B_eq_* = *B_max_* × ([*Ab*]/([*Ab*] + *K_D_*), (1)

where *B_eq_* is the response value at equilibrium and [*Ab*] is the concentration of anti-CD44 antibodies. Approximation was performed for each antibody concentration using Kinetic Evaluation (Metrohm Autolab BV, Leusden, The Netherlands).

The SPR response of the interaction of QDs-anti-CD44 conjugates with immobilized CD44 was calculated as the maximum capacity of Au/CD44/QDs-anti-CD44 on the surface under steady-state conditions [51]. The response was determined by fitting the QDs-anti-CD44 association phase dataset to the hyperbolic function y = *a*x/(*b* + x), where parameter *a* is the equilibrium angle [m°]. Error bars were calculated as the standard deviation of 3–5 independent measurements.

## 4. Conclusions

In this study, we propose using SPR spectroscopy to evaluate the conjugation efficiency of QDs and antibodies. Monitoring the interaction of conjugates via the binding of bio-recognition molecules with immobilized specific antigen can provide information on optimal conjugation conditions. An initial molar QDs-NH_2_: anti-CD44 ratio of 1:10 was shown to be optimal for conjugation using BS3 cross-linking agent. Moreover, it was shown that the conjugates prepared using optimal molar QDs-NH_2_: anti-CD44 ratio specifically bound to immobilized CD44 protein. Optimizing their conjugation affects the sensitivity of analytical systems based on conjugates of QDs and antibodies. The proposed SPR application can be used to improve analytical parameters of such systems.

The same evaluation procedure can be used to optimize the conjugation of antibodies or other bio-recognition molecules with QDs or other nanostructures, such as metal nanoparticles, magnetic nanoparticles, etc. Moreover, the proposed method can be applied to compare immobilization strategies or to select the best antibodies for conjugation. However, it should be noted that there are a number of limitations regarding SPR measurements. Unbound antibodies must be removed from the conjugate solution before performing measurements. Their presence in the solution leads to inaccurate data and possible erroneous estimation, since antibodies can interact with immobilized specific antigen. Additionally, proper surface regeneration of the SPR disk is critical for the reusability required to automate the process. It is recommended to confirm conjugation by other methods such as DLS or electrophoresis before performing SPR measurements. SPR spectroscopy is suitable for sensitive real time interaction monitoring, but cannot provide information on which molecules or particles bind to the surface.

## Figures and Tables

**Table 1:** Hydrodynamic diameter of unmodified and modified QDs measured using DLS.

	Hydrodynamic Diameter, *D_H_* [nm]
QDs-COOH	29.54 ± 1.25
QDs-NH_2_	30.21 ± 1.80
Anti-CD44	12.98 ± 1.08
QDs-anti-CD44 1:3	35.31 ± 1.14
QDs-anti-CD44 1:5	38.09 ± 1.62
QDs-anti-CD44 1:10	46.63 ± 3.86
QDs-anti-CD44 1:12	42.42 ± 0.80

## Data Availability

The data presented in this study are available on request from the first author.

## Supplementary Materials

The following supporting information can be downloaded at: https://www.mdpi.com/article/10.3390/ijms232012626/s1.
