# Supplementary material for: Experimental Evaluation of Quantum Dots and Antibodies Conjugation by Surface Plasmon Resonance Spectroscopy
Source: Int J Mol Sci. 2022 Oct 20;23(20):12626. doi: 10.3390/ijms232012626 (PMC9603974; doi:10.3390/ijms232012626)
Supplement: Supplementary file 1 [file ijms-23-12626-s001.zip › ijms-1936826-supplementary.pdf]

Experimental Evaluation of Quantum Dots and Antibodies Conjugation by Surface Plasmon Resonance Spectroscopy

Anton Popov [1,2*], Viktorija Lisyte [2], Asta Kausaite-Minkstimiene [1,2], Eiva Bernotiene [3,4], Almira Ramanaviciene[1,2*]

[1] Department of Immunology, State Research Institute Centre for Innovative Medicine, Santariskiu Str. 5, LT-08406 Vilnius, Lithuania
[2] NanoTechnas—Center of Nanotechnology and Materials Science, Institute of Chemistry, Faculty of Chemistry and Geosciences, Vilnius University, Naugarduko Str. 24, LT-03225 Vilnius, Lithuania
[3] Department of Regenerative Medicine, State Research Institute Centre for Innovative Medicine, Santariskiu Str. 5, LT-08406 Vilnius, Lithuania
[4] Department of Chemistry and Bioengineering, The Faculty of Fundamental Sciences, Vilnius Gediminas Technical University, Vilnius-Tech, LT-10223 Vilnius, Lithuania

* Correspondence: anton.popov@chgf.vu.lt (A.P.); almira.ramanaviciene@chf.vu.lt (A.R.)

Supplementary data

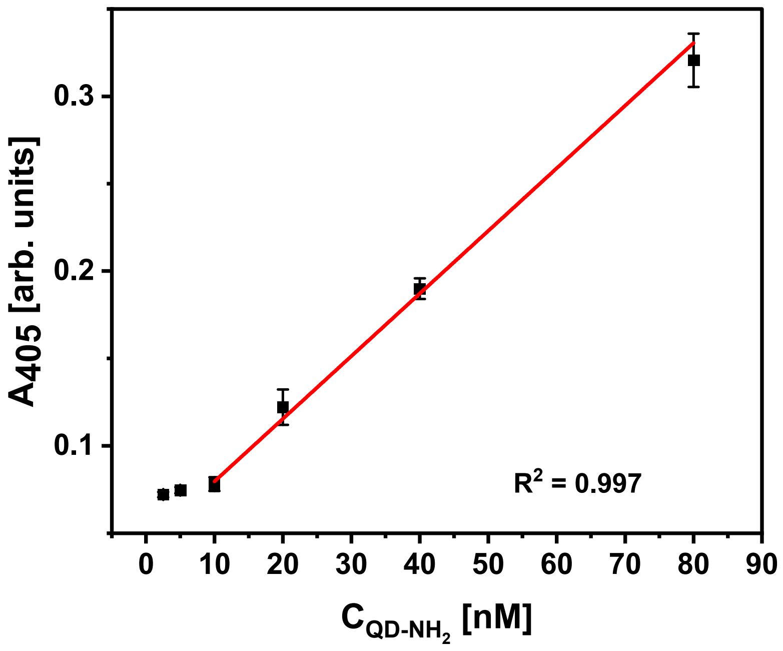

Figure S1. Calibration curve of QDs-NH_2 present in the solution.

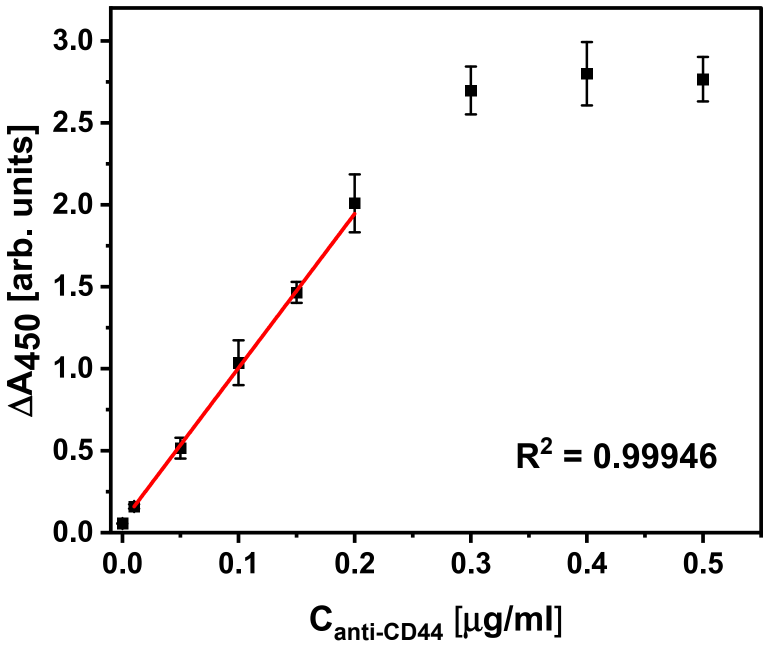

Figure S2. The calibration curve for detection of anti-CD44 antibody using direct ELISA format.

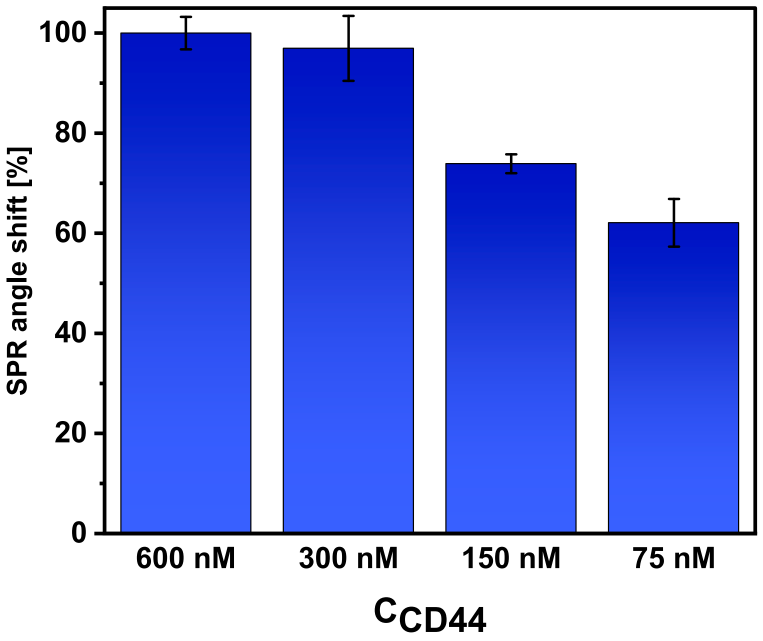

Figure S3. Interaction of QDs-NH_2 : anti-CD44 conjugates with CD44 biomarker, immobilized on the surface of sensor disk using initial CD44 concentrations of 75, 150, 300, and 600 nM.
